# Elevating Public Health through European Elections: A Call to Action

**DOI:** 10.1093/eurpub/ckae006

**Published:** 2024-02-05

**Authors:** Charlotte Marchandise, Hans Henri Kluge, Floris Barnhoorn, Ricardo Mexia

**Affiliations:** Executive Director, EUPHA; WHO/Europe Regional Director; WHO/Europe Regional Director; WHO/Europe Regional Director

As we stand at the crossroads of political decision-making in Europe, it is imperative to reflect on the profound impact that elections have on the health and well-being of our continent. The upcoming European elections are not just a political event; they are a pivotal moment for public health, demanding our collective attention and advocacy.

At the European Public Health Association EUPHA, our commitment to fostering a healthier Europe is unwavering. Our mission goes beyond the confines of research and academia; it extends into the realm of policy and decision-making. The European elections present a unique opportunity for us to assert the importance of evidence-based public health policies in shaping the future of our continent. This is not something we will do on our own: a campaign is about to be launched by the EU4Health Civil Society Alliance with a EU elections 2024 manifesto and shared priorities to secure Health on the political agenda.

As executive director of EUPHA, and from my former functions, I am acutely aware of the intricate relationship between political decisions and the health outcomes of our diverse population. Our responsibility is not merely to observe from the sidelines but to actively engage in the democratic process, ensuring that the voices of public health professionals resonate in the corridors of power.

The nexus between politics and public health cannot be overstated. Policies enacted at the European level have far-reaching consequences for the health of millions. From environmental regulations to healthcare accessibility, each decision holds the potential to either nurture or undermine the well-being of our communities. All of our members can be part of this campaign to amplify it by contacting local and national policy makers in their national networks.

EUPHA is committed to being a of evidence-based advocacy. We will leverage our expertise to provide policymakers with the necessary data and insights to inform decisions that prioritize health and equity. New ways of working will help us to bridge the gap between research and policy; the leadership of young professionals with EUPHAnxt is truly inspiring, and no doubt they will be able to commit to this campaign.

Together, we can ensure that the European elections become a catalyst for positive and equitable change, paving the way for a healthier and more resilient continent.

## Sailing the waves of European public health: exploring a sea of innovation

BarnhoornFloris
WHO/Europe Regional DirectorMexiaRicardo
WHO/Europe Regional Director

### Welcome to Lisbon 2024

After the successful conference in Dublin, we will meet again in person for the 17th EPH Conference in Lisbon, Portugal, from 12–15 November. The charming city of Lisbon is the perfect blend of historical charm and modern innovation, making it an ideal location for exploring cutting-edge public health topics. We will meet in the renovated Lisbon Congress Center (CCL), a venue with excellent facilities and sustainable features. Located near the iconic 25 de Abril Bridge and the Belém Tower, the CCL offers easy access to the city’s most famous landmarks and cultural institutions.

### Abstract submission for Lisbon 2024

Abstract submission is open from 1 February to 1 May 2024. EPH Conferences offer excellent opportunities to learn from the latest research and practice, to network with experts and colleagues and to expand your professional horizons.

Abstracts are invited for workshops and single presentations in all areas of public health research, practice, policy and education. For less experienced abstract submitters, a Tutoring Programme is set-up. Check our website for regular updates.

### Sailing the waves of European public health: exploring a sea of innovation

The theme for Lisbon 2024 is *Sailing the waves of European public health: exploring a sea of innovation.* Just as explorers left Lisbon in search of new worlds, we, as public health professionals, embark on a journey of discovery in our field. During the conference we aim to push the boundaries, discover new approaches and chart a new course for the future of public health in Europe.

The plenary programme is centred on five subthemes:

Artificial intelligence (AI) in Public Health: we’ll explore how AI is transforming disease surveillance, healthcare delivery, and decision-making, propelling us into an exciting era of health innovation.

One Health—Animal Approach: we’ll discuss how taking an animal-centric approach can strengthen public health strategies and mitigate emerging threats.

Social Marketing in Public Health: join us in exploring innovative campaigns, digital strategies, and community engagement techniques to promote healthier behaviours.

Health Inequalities: the diverse neighbourhoods of Lisbon, each with its own character and challenges, serve as a reminder of the widespread health inequalities in Europe that persist across the continent. There will be a discussion on innovative strategies that can be employed to reduce disparities and ensure equitable access to health care.

Global Health: we’ll discuss how European public health efforts can contribute to global health solutions, addressing issues that transcend borders.

The plenary programme is organized by: EPH Conference Foundation, EUPHA, EuroHealthNet, European Health Management Association (EHMA), ASPHER, European Observatory on Health Systems and Policies, European Centre for Disease Prevention and Control (ECDC), European Commission, WHO Regional Office for Europe. Check our **website** for regular updates.

### Conference fees

Conference fees will be announced in March. Registration is possible for the main conference (13—15 November) and pre-conferences (12 November). Day registration is also an option. Registration fees include buffet lunches and refreshments in morning and afternoon. Reduced rates are available for EUPHA members, delegates from Portugal, students/trainees and delegates from LMICs.

We look forward to seeing you all in Lisbon.

